# Untargeted metabolomics unveils metabolic biomarkers in HFpEF

**DOI:** 10.3389/fmolb.2025.1673430

**Published:** 2025-10-21

**Authors:** Dongqin Duan, Muyashaer Abudurexiti, Refukaiti Abuduhalike, Salamaiti Aimaier, Ailiman Mahemuti

**Affiliations:** Department of Heart Failure, First Affiliated Hospital of Xinjiang Medical University, Urumqi, China

**Keywords:** HFPEF, metabolomics, tryptophan metabolism, indole, kynurenine, biomarker

## Abstract

**Background:**

Heart failure with preserved ejection fraction (HFpEF) is a complex condition linked to metabolic disturbances. This study aimed to identify plasma metabolic signatures in HFpEF patients using untargeted metabolomic profiling.

**Methods:**

We analyzed data from 30 HFpEF patients and 30 matched healthy controls. Untargeted metabolomic profiling via UHPLC-MS/MS was conducted on venous blood to identify metabolic differences. Initial analyses included principal component analysis (PCA), partial least squares-discriminant analysis (PLS-DA), and hierarchical clustering to detect differing compound groups. Receiver operating characteristic (ROC) curve analysis and pathway enrichment were performed to identify dysregulated genes. Finally, enzyme-linked immunosorbent assay (ELlSA) was used to validate the serum levels of selected metabolites.

**Results:**

A total of 124 significantly different metabolites were identified (VIP >1.0, FC > 1.2 or <0.833, P < 0.05). Lipids and lipid-like molecules were notably altered in HFpEF patients. KEGG enrichment analysis indicated these metabolites were primarily involved in tryptophan metabolism. Hierarchical clustering showed distinct compound levels between groups. ROC curve analysis revealed PC 18:1-20:5 (AUC: 0.833) and PC 18:1-18:1 (AUC: 0.824) as key metabolites. ELlSA validation confirmed that serum Kynurenine and IAA levels were significantly elevated in HFpEF patients compared to HCs (p < 0.05).

## 1 Introduction

Heart failure (HF) is a complex syndrome and often the end-stage of various cardiovascular diseases. Heart failure with preserved ejection fraction (HFpEF) is a subtype that affects up to half of the approximately 65 million HF patients worldwide (Hahn et al., 2023). However, its pathophysiology remains poorly understood. Among the diverse factors contributing to HFpEF, metabolic disturbances have emerged as critical elements that influence the disease trajectory (Ferro et al., 2022). These disturbances reflect a complex interplay between genetic predispositions, comorbid conditions, and environmental factors, ultimately affecting cardiac and systemic homeostasis (Henkens et al., 2020).

Metabolic alterations have been implicated in the development and progression of various cardiovascular diseases, including heart failure (Pieske et al., 2020; Anker et al., 2023; Hunter et al., 2016)^.^ Nonetheless, the majority of these studies utilized animal models, leading to a paucity of data on human HFpEF metabolism. Concurrently, interest in metabolic impairment as a potential contributing factor to the onset and progression of HFpEF has increased (Hahn et al., 2023; Schiattarella et al., 2019; Palazzuoli et al., 2023; Bekfani et al., 2022).Therefore, investigating the plasma metabolic profile of HFpEF patients could provide valuable insights into the underlying mechanisms and potentially identify novel biomarkers for early diagnosis and targeted therapies (Zordoky et al., 2015).

In recent years, developments in analytical techniques, particularly ultrahigh-performance liquid chromatography coupled with tandem mass spectrometry (UHPLC-MS/MS), have greatly enhanced the comprehensive and accurate profiling of metabolites in biological samples.High sensitivity, selectivity, and throughput are provided by UHPLC-MS/MS, rendering it an ideal platform for metabolomic analysis (Deng et al., 2022; Dunn et al., 2011). This study employed untargeted metabolomics to compare metabolite expression profiles between HFpEF patients and healthy controls to uncover new insights and potential therapeutic targets for HFpEF.

## 2 Materials and methods

### 2.1 Participants and clinical sample collection

HFpEF Study Population: Samples were collected from 30 HFpEF patients and 30 healthy controls at the First Affiliated Hospital of Xinjiang Medical University between 1 March 2023, and 31 July 2023. All adult subjects provided written informed consent to participate in the study.

Diagnosis Criteria: Patients were diagnosed with HFpEF based on the following consensus criteria (Pieske et al., 2020; Anker et al., 2023; McDonagh et al., 2021): symptoms and signs of exertional dyspnea (New York Heart Association class II or III), HF with left ventricular ejection fraction (LVEF) ≥ 50%, and at least two of the following: (1) elevated NT-proBNP (N-terminal pro-B-type natriuretic peptide) ≥125 pg/mL; (2) structural heart disease or diastolic dysfunction on echocardiography; and (3) E/e’ ≥9.

Exclusion criteria: Patients with a history of congenital heart disease, LVEF <40%, HF with mid-range EF (40%–50%), hypertrophic cardiomyopathy, cardiac transplantation, constrictive pericarditis, severe valvular disease, or infiltrative or restrictive cardiomyopathy were excluded (Hahn et al., 2023; Hahn et al., 2021).

Ethical Approval: This study received approval from the Ethics Committee of the First Affiliated Hospital of Xinjiang Medical University. Informed consent was obtained from each participant.

Sample collection: Overnight fasting venous blood samples were collected in the morning before breakfast. After centrifugation at 3,000 rpm for 10 min, the supernatant was collected and stored at −80 °C until analysis.

### 2.2 Plasma sample preparation

Sample Processing: Plasma samples obtained in EDTA tubes were promptly processed. Each 100 μL sample was resuspended in pre-chilled 80% methanol and then vortexed thoroughly. After 5 min of incubation on ice and 20 min of centrifugation at 15,000 × g at 4 °C, the supernatants were collected and diluted with LC-MS grade water to achieve a final concentration of 53% methanol.

LC‒MS/MS analysis: The diluted samples were further centrifuged for 20 min at 15,000 × g and 4 °C. The supernatants were then subjected to LC‒MS/MS analysis.

Quality Control: Quality control (QC) samples, comprising equal volumes of mixtures of experimental samples, were prepared to monitor the chromatography‒mass spectrometry system balance, system stability, and instrument status throughout the experiment. Blank samples were also added to remove background ions.

### 2.3 UHPLC-MS/MS analysis

The plasma samples were analyzed by UHPLC-MS/MS on a Vanquish UHPLC system (Thermo Fisher, Germany) coupled to either an Orbitrap Q Exactive HF or an Orbitrap Q Exactive HF-X mass spectrometer (Thermo Fisher, Germany) at Novogene Co., Ltd. (Beijing, China). Samples were injected onto a Hypersil Gold column(100 × 2.1 mm,1.9 μm)and analyzed at a flow rate of 0.2 ML/min over a 12-min linear gradient. The positive ion mode eluents included 0.1% formic acid in water (eluent A) and methanol (eluent B), while the negative ion mode eluents consisted of 5 mM ammonium acetate (pH 9.0, eluent A) and methanol (eluent B). The elution profile was as follows: 1.5 min with 2% B; 3 min with 2%–85% B; 10 min with 85%–100% B; 10 min with 100%–2% B; and 12 min with 2% B. The Q Exactive™ HF mass spectrometer was operated under the following conditions: positive/negative ion mode, 3.5 kV spray voltage, 320 °C capillary temperature, 350 °C aux gas heater temperature, 10 L/min aux gas flow rate, 35 psi sheath gas flow rate, and an S-lens RF level of 60.

### 2.4 Data processing and metabolite identification

The raw data from UHPLC-MS/MS were processed using Compound Discoverer 3.3 (CD3.3, Thermo Fisher) for peak alignment, picking,and quantitation. Key parameters included: peak area correction with the first QC, mass tolerance of 5 ppm, signal intensity tolerance of 30%, and minimum intensity. Peak intensities were then adjusted to the total spectral intensity. This normalized data was used to predict molecular formulas based on additive ions, molecular ion peaks, and fragment ions. Peaks were matched with mzCloud (https://www.mzcloud.org/), mzVault,and Mass List databases for correct qualitative and relative quantitative results. Statistical analyses were conducted using R (R version R-3.4.3), Python (version 2.7.6), and CentOS (release 6.6). For non-normally distributed data, relative peak areas were standardized using the formula: raw quantitation value/(sum of sample metabolite quantitation/sum of QC1 metabolite quantitation). Compounds with CVs of relative peak areas in QC samples exceeding 30% were excluded, leading to the final identification and relative quantification of metabolites.

### 2.5 Data analysis

Metabolite annotation in plasma samples was performed with the KEGG (https://www.genome.jp/kegg/pathway.html), LIPIDMaps (http://www.lipidmaps.org/) and HMDB (https://hmdb.ca/metabolites) databases. Using metaX, partial least squares discriminant analysis (PLS-DA) and PCA were conducted. Univariate regression (t-test) was used to determine significant differences (P value). Metabolites meeting the criteria of VIP >1 and P value <0.05 and fold change >1.2 or FC < 0.833 were classified as differentially expressed. Volcano plots generated by ggplot2 in R facilitated the selection of metabolites based on log2(FC) and -log10(P value).

### 2.6 Enzyme-linked immunosorbent assay (ELISA) for clinical blood samples

Serum kynurenine and Indole-3-acetic acid levels were measured in a total of 78 participants, including 38 healthy controls and 40 patients newly diagnosed HFpEF. All blood samples were collected after an overnight fast, centrifuged at 3000 *g* for 10 min, and the supernatants were stored at −80 °C for subsequent analysis. Serum levels were measured by ELISA kit Kynurenine (Human kynurenine ELISA Kit YS04739B Yaji-Biotechnology), Indole-3-acetic acid (Human Indole 3-acetic acid ELISA Kit L0511 Yaji-Biotechnology), according to the manufacturer’s instructions.

## 3 Results

### 3.1 Baseline features of participants

Table 1 compares the basic characteristics of the HFpEF and HC groups. The groups were matched for age and sex. Compared with the two groups, the HFpEF group presented significantly greater levels of IL-6, LP(b), NT-ProBNP, and Cr and lower levels of TC, LDL, and HDL, with notable incidences of hypertension (70%, *p* = 0.004), CAD (56.7%, *p* < 0.001), and DM (46.7%, *p* < 0.001). However, lower levels of TC and LDL in HFpEF patients might be attributable to lipid-lowering treatments. Other parameters, such as CRP, TG, LP(a), E/e`, LVED, and SAA, were not significantly different between the groups (P > 0.05).

**TABLE 1:** Basic characteristics of the participants.

Variables	HFpEF (n = 30)	HC (n = 30)	P Value
Male, n (%)	17 (56.7%)	15 (50%)	0.605
Female, n (%)	13(43.3%)	15(50%)	0.420
Age (years)	61.47 ± 11.87	52.57 ± 15.34	0.490
BMI ((kg/m2)	25.33 ± 4.18	24.63 ± 3.52	0.500
LVEF, %	61.01 ± 3.65	62.80 ± 2.07	0.023
CRP	10.04 ± 6.29	7.68 ± 2.10	0.057
IL-6	3.90 (2.83, 6.72)	2.35 (1.95, 3.71)	0.003
TC ((mmol/L)	3.10 ± 0.69	4.36 ± 1.63	<0.001
LDL (mmol/L)	1.90 ± 0.51	2.77 ± 0.90	<0.001
HDL (mmol/L)	0.92 ± 0.25	1.10 ± 0.26	0.011
TG (mmol/L)	0.9 (0.64, 1.21)	1.04 (0.72, 1.78)	0.297
LP(a)	240.14 (85.73, 706.13)	122.88 (25.37, 493.50)	0.081
LP(b)	0.68 ± 0.15	0.10 ± 0.37	<0.001
NT-ProBNP	433.5 (162.75, 1192.5)	45.65 (24.83, 69.85)	<0.001
Cr	68.92 (59.45, 84.84)	58.04 (47.67, 78.63)	0.048
E/e`	8.64 ± 2.52	8.35 ± 2.41	0.649
LVED	49.17 ± 5.57	47.40 ± 2.40	0.116
SAA	4.34 (1.96, 6.28)	3.225 (2.48, 4.20)	0.141
Hypertension, n (%)	21 (70)	10 (33.3)	0.004
CAD, n (%)	17 (56.7)	5 (16.7)	0.001
DM, n (%)	14 (46.7)	2 (6.7)	<0.001
AF, n (%)	2 (6.7)	0	0.472
PAH, n (%)	3 (10)	0	0.236
Lipid-lowering agents	19 (63.3)	11 (36.7%)	<0.01
ACEI/ARB	4 (13.3)	2 (6.7)	<0.01
ARNI	2 (6.7)	0 (0)	<0.01
Beta-blocker	4 (13.3)	3 (10)	<0.01

BMI, body mass index; LVEF, left ventricular ejection fraction; CRP, C-reactive protein; IL6, interleukin 6; TC, total cholesterol; LDL, low-density lipoprotein cholesterol; HDL, high-density lipoprotein cholesterol; TG, triglyceride; NT-proBNP, N-terminal pro-B-type natriuretic peptide; Cr, creatinine; SAA, serum amyloid A protein; CAD, cardiovascular disease; DM, diabetes mellitus; AF, atrial fibrillation; PAH, pulmonary arterial hypertension, ACEI, angiotensin-converting enzyme inhibitor; ARB, angiotensin receptor blocker; ARNI, angiotensin receptor neprilysin inhibitor.

### 3.2 Quality control of untargeted metabolic profiling

**FIGURE 1:**
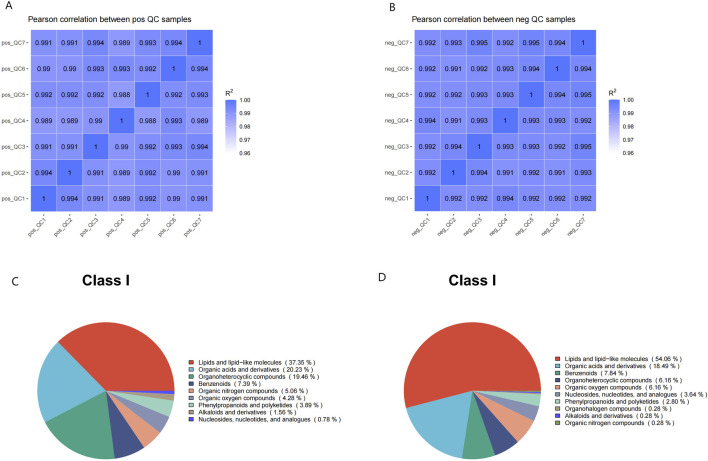
The quality control of untargeted metabolomic profiling. **(A,B)** Pearson correlation analysis between QC samples: the coefficient (R2) values were both nearly 1 under the positive **(A)** or negative **(B)** polarity modes. **(C,D)** Metabolite class I categorical pie chart in the positive **(C)** and negative **(D)** ion modes.

Considering the influence of exogenous factors on the metabolome, ensuring instrumental stability and a normal signal response during metabolite detection, critical QC was performed via Pearson correlation analysis between the QC samples and principal component analysis (PCA). Pearson correlation coefficients (R2) among the QC samples were close to 1 in both ion modes (Figures 1A,B), indicating high stability and data quality.

### 3.3 Metabolite pathways and classification annotations

**FIGURE 2:**
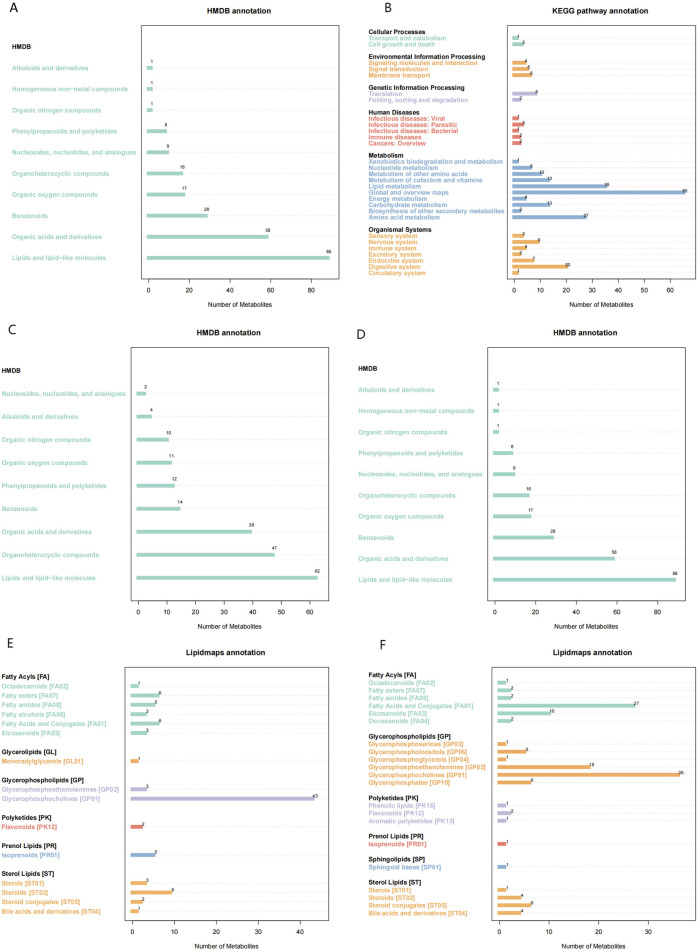
Metabolic Pathways and Classification Annotations **(A,B)** KEGG Pathway Annotation in the positive **(A)** and negative **(B)** ion modes. **(C,D)** HMDB classification annotation in the positive **(C)** and negative **(D)** ion modes. **(E,F)** LIPID MAPS classification annotation in the positive **(E)** and negative **(F)** ion modes.

Comparative analysis of metabolic signatures in untargeted metabolomics between the HFpEF and HC groups under the ESI+ and ESI− modes. The statistical analysis of the identified chemically classified metabolites revealed that lipid metabolites constituted the majority in both the positive and negative ion modes, accounting for 37.35% and 54.06%, respectively (Figures 1C,D). Subsequently, to understand the functional characteristics and classification of different metabolites, metabolite pathway, and classification annotations were performed using major databases such as KEGG, HMDB, and LIPID MAPS (Figures 2A–F).

### 3.4 Identification of differentially expressed metabolites

**FIGURE 3:**
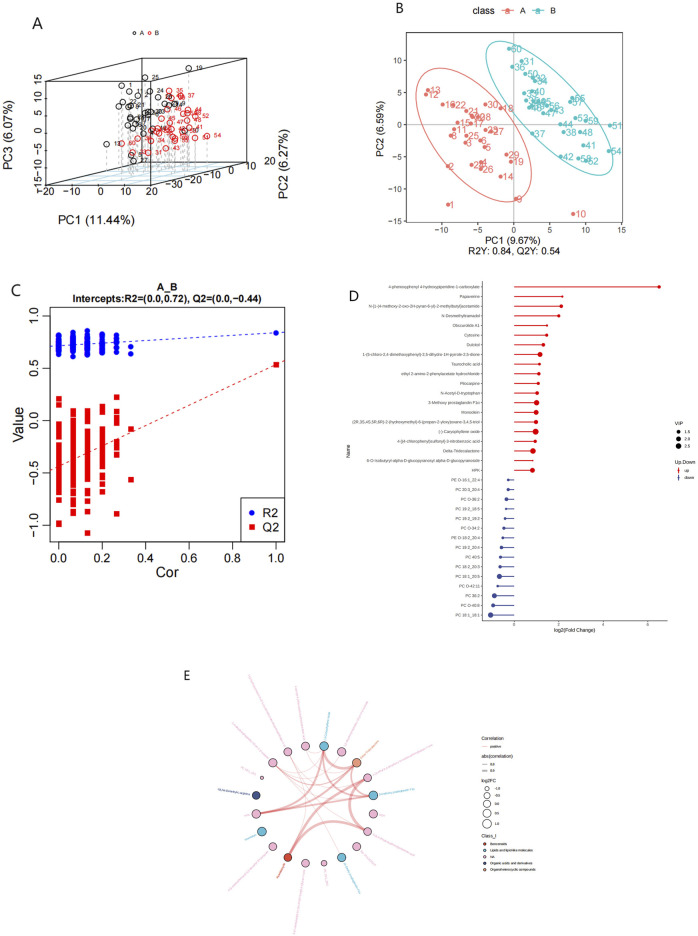
Metabolic alterations in HFpEF and HC samples. PCA 3D score plot for the data from HFpEF patients (black) and HCs (red) **(A)**. PLS-D analysis of differentially abundant metabolites in the HFpEF and HC groups **(B)** and cross-validation plot **(C)** with a permutation test repeated 200 times. **(D)** Lollipop chart **(D)** illustrating DEMs between the HFpEF and HC groups. **(D)** Hierarchical cluster analysis of DEMs between HFpEF patients and HCs. **(E)** Chord diagram **(E)** showing interaction of metabolites between HFpEF group and the HC group. Widths correspond to interaction magnitude; colors encode correlation.

The investigation of alterations in various metabolites within the HFpEF group necessitated the application of multivariate statistical methodologies, specifically PCA and PLS-DA, to elucidate the relationship between biological features and metabolomics. Unsupervised PCA, a conventional approach in pattern recognition, was employed to scrutinize the distribution of the HFpEF group and remove outlier data. PCA revealed the distinctiveness of the two groups based on PC1 and PC2. Concurrently, disparities in metabolite profiles between the HFpEF and HC groups were observed (Figure 3A). Furthermore, supervised PLS-DA multivariate analysis corroborated significant differences between the two groups, revealing distinct clustering of the HFpEF and HC groups (Figure 3B). Additionally, the results of the permutation test strongly indicated that the original model was valid (R2 intercept = 0.72, Q2 intercept = −0.44, Figure 3C), suggesting that the PLS-DA model did not overfit. These results indicate a favorable model fit and predictive performance.

To elucidate the characteristics of plasma metabolites within the HFpEF group and identify metabolites confidently associated with HFpEF, distinctions between the ESI+ and ESI- modes were made based on VIP (variable importance in projection, VIP) > 1.0, FC > 1.2 or FC < 0.833 and a P value <0.05. Overall,993 differential compounds were identified from plasma samples, 124 of which reached statistical significance. Among these, 87 metabolites exhibited upregulation, with fold changes reaching up to 2.77, and 15 metabolites displayed downregulation, with fold changes as low as 0.48. A lollipop chart was generated to visualize the distribution of the top 20 differentially expressed metabolites (DEMs) between the two groups (Figure 3D). Notably, the downregulation of specific phospholipids may point to altered lipid metabolism and energy use in HFpEF patients. Additionally, a chord diagram was constructed to depict the significantly different plasma metabolites between the HFpEF group and the HC group (Figure 3E). Notably, the significantly upregulated metabolites in the HFpEF group included amino acids, whereas the significantly downregulated metabolites included phosphatidylcholines (PCs) and phosphatidylethanolamines (PEs) (Supplementary Table S1).

### 3.5 Significance of differentially abundant metabolites in diagnosing HFpEF

**FIGURE 4:**
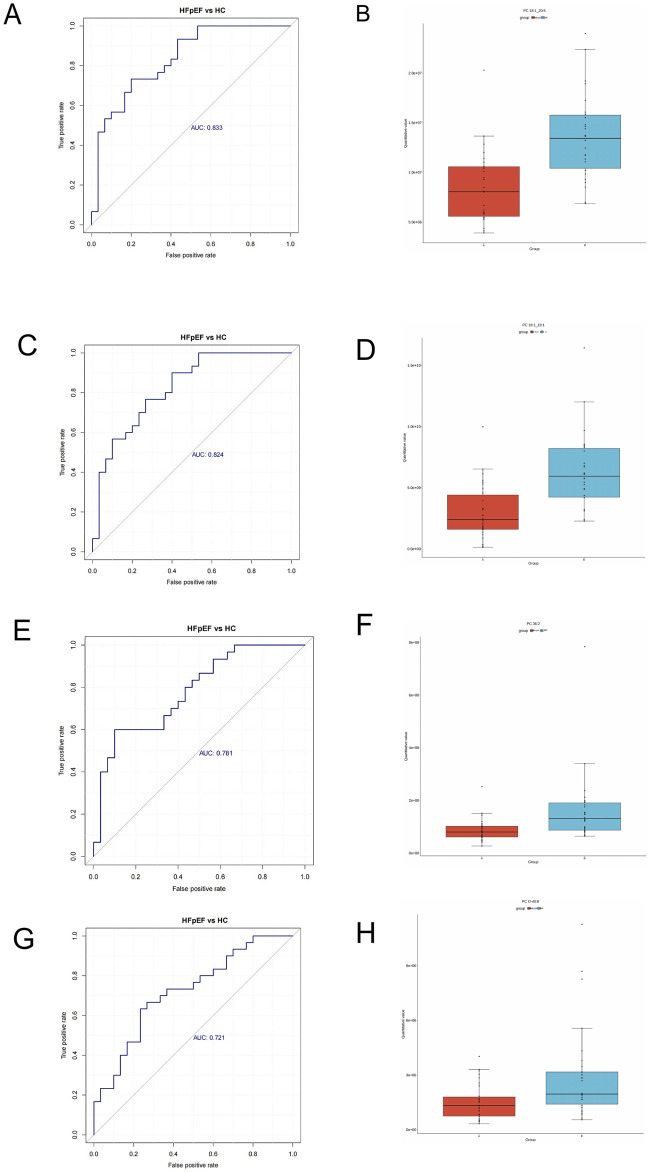
Receiver operating characteristic (ROC) analysis of different metabolites. **(A)** PC 18:1_20:5. **(C)** PC 18:1_18:1. **(E)** PC 36:2, **(G)** PC 0-40:8. Box plots of selected DEM concentrations between HFpEF patients (red) and HCs (green). **(B)** PC 18:1_20:5. **(D)** PC 18:1_18:1. **(F)** PC 36:2, **(H)** PC 0-40:8.

Univariate ROC curves were generated for each metabolite to assess their diagnostic potential for HFpEF. In this investigation,As shown in Figures 4A,C,E, G that PC 18:1-20:5 (AUC:0.833), PC 18:1-18:1 (AUC:0.824), PC 36:2 (AUC:0.781), and PC 0-40:8 (AUC:0.721) could serve as biomarkers for HFpEF. These metabolites also exhibited the highest significance, with PC 18:1-20:5 (AUC:0.833) and PC 18:1-18:1 (AUC:0.824) acetate demonstrating superior area under the curve (AUC) values. Furthermore,the expression levels of these metabolites were much lower than those in the HC group (Figures 4B,D,F,H), This might suggest a significant difference in the metabolism of this lipid component between the groups.

### 3.6 Pathway enrichment analysis

**FIGURE 5:**
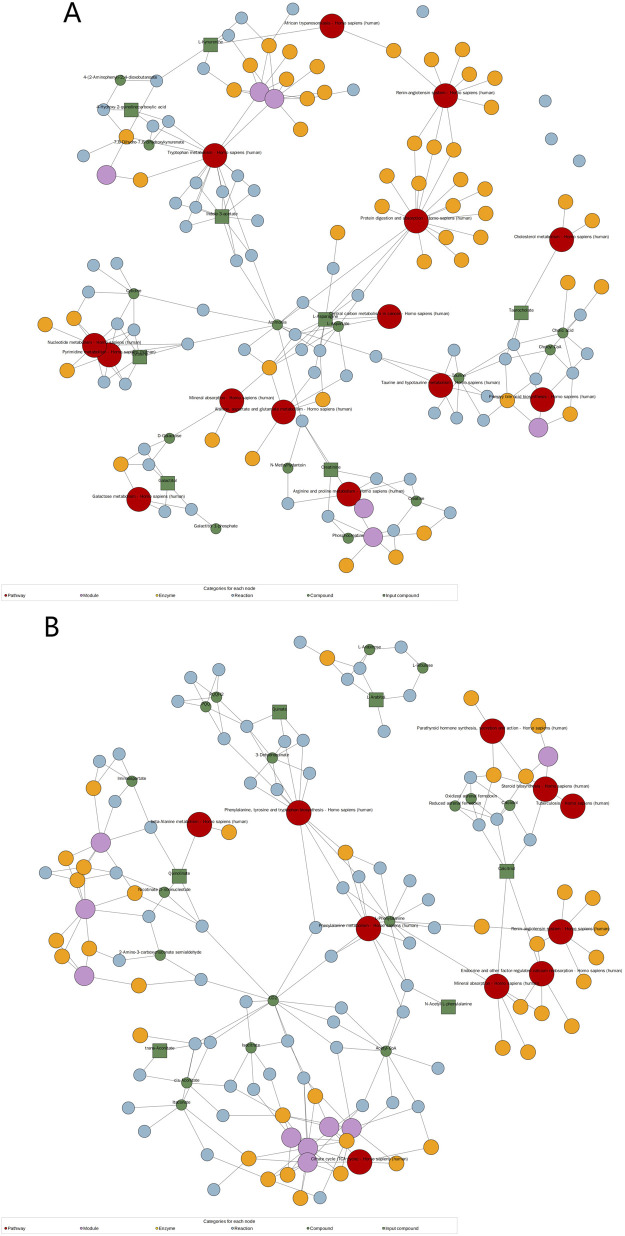
Significantly changed pathways based on the enrichment analysis. KEGG network diagram positive **(A)** and negative **(B)** ion modes. (Note: Red dots represent a metabolic pathway, yellow dots represent information about a substance-related regulatory enzyme, green dots represent a background substance of a metabolic pathway, purple dots represent information about a class of substance molecular modules, blue dots represent a chemical interaction reaction of a substance, and green squares represent differential substances obtained in this comparison).

Analysis of the KEGG network diagram (Figure 5) revealed prominent activation of the tryptophan (Trp) pathway in the examined plasma samples. Compared with those in healthy controls, the levels of key regulatory metabolites within this pathway, such as indole-3-acetate and L-kynurenine, were significantly increased (p < 0.05). These metabolites are shown in green in the network diagram, signifying their elevated expression. The Trp pathway is suggested by these results as a possible therapeutic target for HFpEF.

### 3.7 Clinical correlations of selected metabolites

**FIGURE 6:**
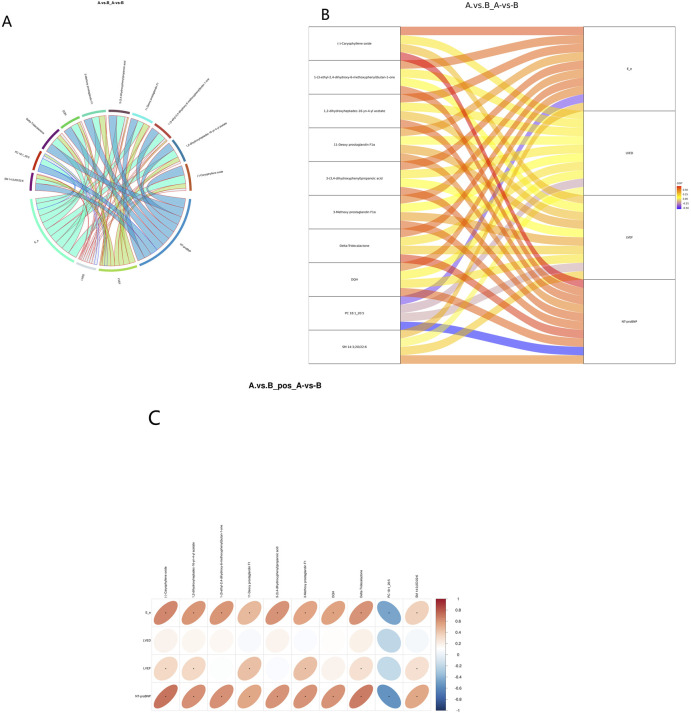
Correlation analysis between DEMs and clinical indicators. **(A)** Correlation chord diagram. (Note: Chord width represents correlation strength; Chord border color stands for correlation, with red and blue representing positive and negative correlations, respectively). Correlation Sankey Diagram Analysis **(B)**. (Note: The left side represents differentially abundant metabolites, while the right side represents NT-pro BNP. The lines represent correlations, with red indicating positive correlation and blue indicating negative correlation). Correlation heatmap **(C)**. (Note: the transverse is the clinical indicators, the longitudinal is the differential metabolite, in the right legend, the correlation coefficient, the red the color, the stronger the positive correlation, the stronger the blue, the stronger the stronger the negative correlation, the higher the ellipse, the absolute value of the correlation, the asterisk (*) in the figure is P < 0.05).

Spearman’s correlation analysis was employed to examine the relationship between metabolites and NT-pro BNP. The 20 most significant metabolites identified through univariate regression demonstrated a moderate-to-high correlation with NT-pro BNP, LVEF, LVED, E/e’ (Figure 6). PC 18:1_20:5 (r = −0.48, p = 0.48) exhibited a predominantly negative correlation with NT-proBNP and E/e’ (Figures 6A–C). This observation suggests that altered lipid metabolism, likely a consequence of metabolic stress, may play a critical role in the disease progression of HFpEF cases.

### 3.8 Validation of tryptophan metabolite alterations in HFpEF patients by ELISA

**FIGURE 7:**
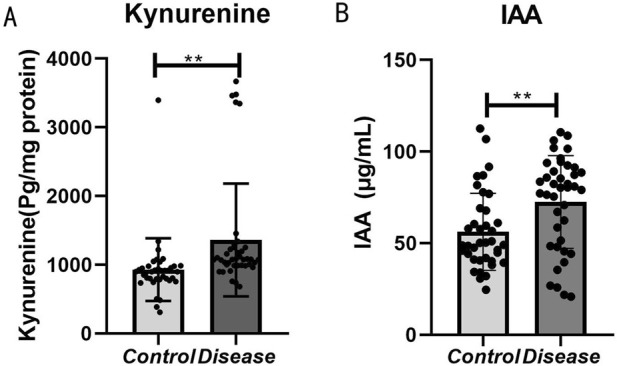
Bar graph comparing kynurenine and IAA levels between control and disease groups. Panel **(A)** shows significantly higher kynurenine levels in the disease group. Panel **(B)** shows higher IAA levels in the disease group. Both panels indicate statistical significance with double asterisks (**).

In the human serum validation conducted via enzyme-linked immunosorbent assay (ELISA), the concentrations of kynurenine (Figure 7A) and indole-3-acetic acid (Figure 7B) were significantly elevated in the HFpEF group. These findings corroborate the metabolomics results, indicating that metabolites along the tryptophan metabolic pathway are markedly altered in patients with HFpEF relative to healthy controls (P < 0.05).

## 4 Discussion

Metabolic impairment significantly influences the onset and progression of HFpEF. However, the detailed metabolic pathogenesis of HFpEF remains largely unexplored. Previous investigations into the metabolic profiles of HFpEF have utilized various metabolomics approaches, including NMR spectroscopy, liquid chromatography‒mass spectrometry (LC‒MS), and gas chromatography‒mass spectrometry (GC–MS) (Zordoky et al., 2015; Bedi et al., 2016; Lanfear et al., 2017; Piatek et al., 2022; Hage et al., 2020). In this study, offers a comprehensive metabolic profiling of HFpEF patients compared to healthy controls, utilizing UHPLC-MS/MS technology. Untargeted metabolomics testing identified differentially abundant metabolite expression profiles in the plasma of HFpEF patients and HC, highlighting distinct plasma metabolic characteristics in HFpEF compared to HC.

Initially, the most notable changes in HFpEF relative to HC were in amino acids, peptides, analogs, and lipids, followed by alterations in organoheterocyclic compounds. KEGG enrichment and pathway impact analysis indicated significant differences between the two groups. Notably, pathways such as Tryptophan metabolism was significantly altered, suggesting potential critical impact on HFpEF progression. Additionally, a total of 124 significantly different metabolites were selected. Among them, the PC 18:1-20:5 and PC 18:1-18:1 compounds exhibited significant metabolic signals in our study, but their stability and predictive ability need to be verified in independent cohorts, different platforms, and longitudinal data.

Notably, pathways such as Tryptophan metabolism was significantly altered, suggesting potential critical impact on HFpEF progression. Additionally,124 significantly different metabolites were selected, which PC 18:1-20:5, PC 18:1-18:1 as potential biomarkers.

### 4.1 Metabolic disturbances in HFpEF patients

HFpEF is associated with oxidative stress and inflammation, impaired lipid metabolism, increased collagen production, disturbed lipid metabolism, and reduced nitric oxide signaling (Hage et al., 2020). Diabetes and obesity are risk factors for HFpEF and contribute to left ventricular (LV) diastolic dysfunction, and cardiac lipotoxicity is believed to play a role in its pathogenic mechanism. Excessive fatty acids (FAs) are stored after consumption (Dunlay et al., 2017; Leggat et al., 2021), and various lipids act as signaling molecules in insulin resistance and inflammatory pathways (Pickens et al., 2017; Kojta et al., 2020), thereby influencing cardiovascular disease onset.

Glycerophospholipids, including PC, PE, and lysophospholipids, are crucial for maintaining cell membrane structure and signal transduction. In HFrEF patients, the serum levels of PC, lysoPC, lysoPE, and other substances significantly decrease, indicating that a disruption in phospholipid metabolism is linked to advanced age, poor clinical conditions, and impaired muscle oxidative metabolism (Marcinkiewicz-Siemion et al., 2018). Recent studies have also revealed a significant decrease in the serum levels of PC and lysoPC metabolites in HFpEF patients (Ferro et al., 2022). Our study revealed notable decreases in glycerophospholipid levels (PC 18:1_20:5, PC 18:1_18:1, PC 36:2, PC O-40:8, PC 19:2_20:4, PC 20:3_20:4, PC 18:2_20:3, PC O-34:2, PC 19:2_19:2, PC 40:5, PC O-42:11, PC 19:2_18:5, PC O-36:2), and PE (PE O-16:1_22:4, PE O-18:2_20:4) levels within the plasma of HFpEF patients compared to those in the plasma of HC. These glycerophospholipids play an important role in maintaining cell membrane integrity and signal transduction, and abnormal levels of these lipids can lead to myocardial metabolic disorders induced by lipotoxicity (Wyant and Moslehi, 2022). Furthermore, our study revealed a significant downregulation of phosphatidylcholines (PCs) and phosphatidylethanolamines (PEs), consistent with previous findings in HFpEF patients (Zordoky et al., 2015; Hage et al., 2020). This finding supports the notion that disturbed lipid metabolism due to metabolic stress is likely crucial for HFpEF progression.

Understanding the complexities of lipid metabolism in HFpEF could provide valuable insights for developing targeted treatments. Strategies focusing on modulating lipid uptake, enhancing lipid oxidation, and restoring mitochondrial function may help mitigate disturbances and improve cardiac function in HFpEF patients.

### 4.2 Tryptophan metabolism as a novel pathway in HFpEF

Tryptophan (Trp), an essential amino acid, serve as a precursor for various biochemical reactions in the human body, including the synthesis of serotonin, glycols, glucocorticoids, and diabetic drugs (Wyant and Moslehi, 2022). Trp metabolism is closely associated with various cardiovascular diseases, with increasing research examining its relationship with Heart Failure (HF) (Gong et al., 2020; Masenga et al., 2023; Ravid et al., 2021).

Trp metabolism primarily involves pathways such as kynurenine (Kyn), 5-hydroxytryptamine, and indole, generating bioactive compounds that regulate functions like metabolism, inflammation, neurological function and immune responses (Xue et al., 2023). The gut microbiota significantly affects Trp metabolism by transforming it into various molecules, including indole and its derivatives (Zhang et al., 2021).

Recent studies have linked disturbances in the Trp metabolism pathway and the resulting Kyn upregulation to myocardial infarction and atherosclerosis (Kowalik et al., 2022; Laurans et al., 2018). Trp is metabolized predominantly via the kynurenine pathway, which is activated by inflammatory cytokines (Liu et al., 2017). HFpEF is characterized by chronic low-grade inflammation, which can exacerbate myocardial stress and endothelial dysfunction. The kyn pathway metabolites have pro-inflammatory and oxidative properties that may exacerbate the cardiovascular burden in HFpEF patients. Research indicates that disruptions in tryptophan metabolism are associated with several cardiovascular risk factors, such as hypertension, atherosclerosis, and diabetes, all of which are prevalent in HFpEF populations (Wyant and Moslehi, 2022). A study suggested that elevated levels of kynurenine pathway metabolites could serve as biomarkers for worsening heart failure symptoms and poor outcomes in HFpEF patients (Hage et al., 2020).

In this study, metabolomic analysis revealed significant alterations in the tryptophan metabolic pathway. Further validation using enzyme-linked immunosorbent assay (ELISA) indicated that serum levels of kynurenine and indole-3-acetic acid (IAA) were markedly elevated in patients with HFpEF. These findings suggest that dysregulation of tryptophan metabolism, particularly the increases in kynurenine and IAA, may play a pivotal role in the pathogenesis and progression of HFpEF.

Kynurenine, a metabolite jointly regulated by the gut microbiota and the immune system, has been extensively documented to be closely associated with vascular inflammation, atherosclerosis, and myocardial hypertrophy (Paeslack et al., 2022; Wang et al., 2023). Mechanistic studies (Shi et al., 2023) have demonstrated that kynurenine can activate the aryl hydrocarbon receptor (AHR), thereby promoting myocardial remodeling and fibrosis. Additionally, kynurenine exhibits prominent pro-inflammatory and vasoregulatory effects, further implicating its involvement in the development of cardiovascular diseases (Wang et al., 2010). Recent research also highlights its role in modulating inflammatory responses in relation to cancer and chronic illnesses, providing multidimensional evidence of its systemic effects (Adams et al., 2012; Cervenka et al., 2017).

As a tryptophan-derived uremic toxin, indole-3-acetic acid (IAA) accumulates in patients with chronic kidney disease and has been linked to impaired cardiovascular function and increased mortality risk (Nayak et al., 2024a). *In vivo* studies, such as those by Nayak et al. (2024b) have demonstrated that IAA induces cardiotoxicity by activating inflammatory pathways and promoting myocardial fibrosis, subsequently impairing cardiovascular performance. The elevated IAA levels observed in HFpEF patients suggest that it may contribute to disease progression via inflammatory and remodeling pathways, intensifying cardiac dysfunction.

Previous investigations have confirmed associations between kynurenine, IAA, and cardiovascular diseases. The potential pathogenic mechanisms may involve kynurenine’s activation of immune-inflammatory pathways, leading to fibrosis and myocardial remodeling, and IAA’s role as a uremic toxin that alters the cardiovascular microenvironment, inducing inflammation and fibrosis, ultimately impairing cardiac function. These metabolites may exert synergistic or interconnected effects in the pathogenesis of HFpEF, influencing disease onset and progression.

Despite existing evidence linking kynurenine and IAA to cardiovascular pathology, their precise mechanistic roles in HFpEF remain to be fully elucidated. Future research employing multi-level *in vivo* and *in vitro* approaches, with larger sample sizes and more targeted analyses, is necessary to validate these findings and clarify the underlying pathogenic mechanisms. Such studies could facilitate the development of these metabolites as biomarkers for early diagnosis and disease monitoring, as well as novel therapeutic targets for HFpEF management.

## 5 Conclusion

In conclusion, metabolomics analyses have identified significant alterations in glycerophospholipid metabolism and the tryptophan pathway in HFpEF, with subsequent validation using enzyme-linked immunosorbent assay (ELISA) confirming elevated levels of kynurenine and indole-3-acetic acid in the serum of HFpEF group. Both glycerophospholipid metabolism and the tryptophan pathway play significant roles in regulating cardiovascular health and disease.

## 6 Study limitations

There were some shortcomings of the current study to be noted. First, the sample sizes of the HFpEF and HC groups were relatively modest, which may affect statistical power and the robustness of the conclusions, and raises the risk of both Type I and Type II errors. Future work should replicate these findings in a large cohort and perform *a priori* sample size calculations to ensure adequate statistical power. Additionally, the metabolomic profile provides an overview of metabolic disturbances potentially influenced by confounders such as acute illnesses, other disease states, and medication usage. Consequently, it remains uncertain whether the metabolic disturbances identified are exclusively associated with HFpEF syndrome. The majority of recent metabolomics research involving heart failure patients relies on blood samples; however, (Hahn et al., 2023), recently reported that changes in plasma metabolite contents between In conclusion, metabolomics analyses have identified significant alterations in glycerophospholipid metabolism and the tryptophan pathway in HFpEF, with subsequent validation using enzyme-linked immunosorbent assay (ELISA) confirming elevated levels of kynurenine and indole-3-acetic acid in the serum of HFpEF group. Both glycerophospholipid metabolism and the tryptophan pathway play significant roles in regulating cardiovascular health and disease. and HFrEF patients may not accurately reflect myocardial metabolic characteristics. This finding underscores the importance of using myocardial tissue directly for metabolomics analysis.

Overall, the small sample size in this study could impact the robustness of our results. The metabolome of each individual is highly sensitive to various endogenous and exogenous factors, such as age, sex, diet, environment, geographical location, genetics, and time of day (Miao et al., 2022). Therefore, future studies should focus on the spontaneous screening of HFpEF patients and the validation of these findings. The current results could be further enriched and corroborated by integrating metabolomics research on plasma and feces.

## Data Availability

The raw data supporting the conclusions of this article will be made available by the authors, without undue reservation.
