# Supplementary material for: Untargeted metabolomics unveils metabolic biomarkers in HFpEF
Source: Front Mol Biosci. 2025 Oct 21;12:1673430. doi: 10.3389/fmolb.2025.1673430 (PMC12583173; doi:10.3389/fmolb.2025.1673430)
Supplement: Supplementary file 1 [file Table1.doc]

**Table S1**

Downregulated metabolites identified from metabolomics profiling.

| Metabolite Name | Mode | FC | P value | VIP | trend |
| --- | --- | --- | --- | --- | --- |
| PC 18:1_20:5 | pos | 0.631 | 2.1713 | 2.18 | down |
| PC 18:1_18:1 | pos | 0.479 | 2.9820 | 2.30 | down |
| PC 36:2 | pos | 0.539 | 0.0001 | 2.07 | down |
| PC O-40:8 | pos | 0.518 | 0.0021 | 1.53 | down |
| PC 19:2_20:4 | pos | 0.672 | 0.0065 | 1.38 | down |
| PC 20:3_20:4 | pos | 0.829 | 0.0096 | 1.28 | down |
| PC 18:2_20:3 | pos | 0.644 | 0.0098 | 1.32 | down |
| PC O-34:2 | pos | 0.725 | 0.0018 | 1.29 | down |
| PC 19:2_19:2 | pos | 0.752 | 0.0174 | 1.16 | down |
| PE O-18:2_20:4 | pos | 0.701 | 0.0197 | 1.18 | down |
| PC 40:5 | pos | 0.652 | 0.0233 | 1.26 | down |
| PC O-42:11 | pos | 0.598 | 0.0234 | 1.13 | down |
| PC 19:2_18:5 | pos | 0.776 | 0.0320 | 1.08 | down |
| PE O-16:1_22:4 | pos | 0.830 | 0.0360 | 1.15 | down |
| PC O-36:2 | pos | 0.785 | 0.0476 | 1.48 | down |

POS, Positive; NEG, Negative; FC, Fold change; VIP, Variable important in projection.
